# Changes in gene expression in potato meristems treated with the sprout suppressor 1,4-dimethylnaphthalene are dependent on tuber age and dormancy status

**DOI:** 10.1371/journal.pone.0235444

**Published:** 2020-07-02

**Authors:** Michael A. Campbell, Carley Gwin, Helen H. Tai, Rachael Adams

**Affiliations:** 1 School of Science, Penn State Erie, The Behrend College,Erie, PA, United States of America; 2 Department of Science, Penn State New Kensington, New Kensington, PA, United States of America; 3 Agriculture and Agri-Food Canada, Fredericton, New Brunswick, Canada; United Arab Emirates University, UNITED ARAB EMIRATES

## Abstract

Commercial storage of potatoes often relies on the use of sprout inhibitors to prolong storage and reduce spoilage. The compound 1,4-dimethylnaphthalene (DMN) has seen increase application as a sprout inhibitor in the potato industry as older chemistries are being phased out. The mode of action of DMN is poorly understood as is the sensitivity of potato tissues to this new class of inhibitor. During storage potato tubers transition from a state of endo-dormant to eco-dormant and it is not known if the DMN response is consistent across this developmental transition. RNA-seq gene expression profiling was used to establish if stored potato tubers (*Solanum tuberosum* cv La Chipper) have differential sensitivity to DMN as tubers age. DMN was applied at three different times during storage; just after harvest when tubers are in endo-dormancy, midwinter at early eco-dormancy, and in spring during late eco-dormancy when sprouting was prevented via exposure to cold storage temperatures. Changes in gene expression were lowest during endo-dormancy while midwinter and spring treatments exhibited a greater and more diverse expression response. Functional analysis of differential gene expression demonstrated gene sets associated with DNA replication, cell division, and DNA methylation are suppressed after DMN treatment. However, gene sets associated with salicylic acid, jasmonic acid, abiotic and biotic stress responses are elevated by DMN only after endodormancy terminates. Gene clusters associated with pathogenesis related proteins PR-4 and PR-5 are also upregulated in response to DMN. These results indicate that DMN sensitivity changes as potato tubers age and transition from endo-dormant to eco-dormant in storage and the overall response is a shift in gene classes that regulate growth and response to stress.

## Introduction

Per ton Potato is among the top five crops produced in the world (http://www.fao.org/3/a-i4691e.pdf). In the United State 63% of harvested potatoes are used for processing (National Potato Council, 2016) and require treatment with compounds to prevent sprouting. One of the most used sprout inhibitors is isopropyl *N*-(3-chlorophenyl) carbamate (CIPC) but its use has raised health and safety concerns resulting in a search for newer approaches to potato storage [1]. The compound 1,4-dimethylnaphthalene (DMN), originally isolated from tubers in storage, has been shown to be an effective inhibitor of sprout growth [2, 3]. DMN is a nonpolar molecule that, when fogged into bins containing potato tubers, can prolong storage by slowing sprouting. DMN has also been shown to be effective when applied to seed potatoes, although some changes in tuber size in the following crop were detected [4]. Previous studies found changes in gene expression in tuber meristems are associated with DMN exposure, but those studies involved a single application of DMN to endo-dormant tubers [5], a situation that does not mimic the multiple applications of DMN that occur during commercial storage. Genetic studies do not support the hypothesis that DMN prolongs a dormant state, but they do show that DMN exposure results in changes in genes associated with the regulation of cell division, suggesting a mechanism for growth suppression [5, 6].

While in storage the physiological condition of potatoes tubers changes temporally as the endo-dormant state terminates and as tubers are held from sprouting by reduced temperatures and/or after application of growth inhibitors such as DMN [7–9]. Thus, as storage continues through the natural endo-dormant cycle the physiologic and genetic response of tubers to growth inhibitors, such as DMN, may change. In this study we examine the changes in transcriptional response of potato tubers to DMN application during storage and the during the transition from endo-dormancy to eco-dormancy.

## Materials and methods

### Plant material

Field grown potato tubers (*Solanum tuberosum* cv La Chipper), not treated with any sprout suppressing chemicals, were a gift from Troyer Farms of Waterford, PA. Tubers were harvested in the years of 2015, 2016 and 2017 for this study. Fall harvested tubers were placed in storage for two to three weeks at 10° C and then transferred to Penn State University for treatment and long-term storage. Tubers were treated with DMN and then stored at Penn State under the following conditions: 8° C, and 90% RH. Every three weeks control and DMN treated tubers were removed from storage and placed at 22° C to determine dormancy status. Termination of dormancy was determined by peeping of tuber meristems within one week at 22° C incubation (Campbell et al., 2010). Samples for expression analysis were taken in the fall (October 2015 and 2016) when tubers were endo-dormant, mid-winter after endo-dormancy was terminated (early eco-dormancy) (January 2015 and 2016), and later in mid-spring termed late eco-dormancy (April 2016 and 2017). Tissues harvest and treated from year 2015–2016 are referred as season one and tissue harvest and treated from 2016 to 2017 are referred to as season 2. Samples for qRT-PCR analysis were from tubers harvested in the fall of 2017, placed in storage until dormancy terminated, and then collected in February of 2018.

### DMN treatments

DMN treatments were conducted in the fall of the harvest year while tubers were in endodormancy, tubers were then retreated with DMN in January at early eco-dormancy and then retreated again with DMN at late eco-dormancy. Each treatment involved eight to ten tubers placed in a single layer at the bottom of a 9.5 Liter BBL GasPak chamber and exposed to either water (control) or DMN at a rate of 22.5 μl per chamber. The chambers were placed at 22° C for two days. Following DMN exposure tuber meristems were harvested using a 1mm micro curette, quick frozen in liquid nitrogen, and stored at -80° C. Two 1 cm plugs were removed from each tuber and placed in 10 ml of 70% reagent alcohol, 30% 2,2,4-trimethylpentane (TMP) and 10 ppm of the internal standard 2-ethylnaphthalene. Samples were then sent to Dichlor Analytical Laboratory (Meridian, ID) for determination of DMN residues on the treated tubers. The DMN residue was detected by adding 0.2 M NaCl followed by analysis of the TMP layer using GC and FID detection. Retreatment of tubers with DMN over a prolonged storage period was conducted to mimic the standard commercial applications used in the potato industry.

### RNA-seq

Meristems stored at -80° C were ground to a powder using liquid nitrogen. Total RNA was extracted using a PureLink RNA Mini Kit (www.thermofisher.com) and quantified on a BioSpecNano (www.ssi.shimadzu.com). Samples were sent to the Penn State University Nucleic Acid Core Facility for Illumina sequencing for year one (2015) and two (2016). RNA quality was measured using an Agilent 2100 Bioanalyzer (www.genomics.agilent.com) prior to library production. Samples were sequenced using an Illumina HiSeq generating 150 bp single-end reads. The first-year analysis was run on four biological replicates for each sample generating a total of 223,784,933 raw reads. The second-year analysis was run on two biological replicates for each treatment with a total of 284,373,978 raw reads. The resulting fastq files were uploaded to Cyverse (Cyverse.org) and mapped to the double haploid potato genome (*Solanum tuberosum* SolTub_ver3) using Tophat2-SE and differential expression of transcripts was determined using Cufflinks v2.2.1a [10]. The data discussed in this publication have been deposited in NCBI's Gene Expression Omnibus [11] and are accessible through GEO Series accession number GSE148637. Genome annotation, GO terms, and the double haploid genome were downloaded from the Spud DB database [12]. Transcripts showing a q value of <0.05 were used to construct gene expression profiles of up and downregulated genes. Venn diagrams representing associated gene expression were created using the software Venn Diagrams (http://bioinformatics.psb.ugent.be/webtools/Venn/)

### Gene ontology (GO) analysis

Log_2_ ratio of DMN treated over control untreated gene expression was calculated after adding 1 to all FPKM values to remove zeros for transcripts showing a q value of <0.05 for each of the three time points (endo-dormant, early eco-dormant and late eco-dormant). Genes with log_2_ > 1 were classified as up-regulated with DMN and those with log_2_ < -1 were classified as down-regulated with DMN for season 1 and season 2 data. The DMN up-regulated genes for both seasons were combined in a single gene list for each time point, the same was done for DMN down-regulated genes. This generated six gene lists: 1. endo-dormant DMN up-regulated, 2. endo-dormant DMN down-regulated, 3. February DMN early eco-dormant up-regulated, 4. February DMN early eco-dormant down-regulated, 5. April DMN late eco-dormant up-regulated and 6. April DMN late eco-dormant down-regulated. The GO terms for each list were analysed using topGO [13]. Enriched GO terms were those where p ≤ 0.01 in the topGO Fisher’s exact test.

### qRT-PCR

Expression of transcripts encoding for PR-4/WIN2 was accomplished using qRT-PCR and transcript specific primers for the gene regions found on chromosome 1 (Table 1).

**Table 1:** Primers designed for qt-PCR analysis of win1 and win2.

Gene	Primer	Sequence
WIN2	Forward	ATTGATCCACGATTCTCACCGTCGTTTGAGCTC
(PGSC0003DMT400050018)	Reverse	TCTACTTGGGATGCTAATAAGCCTTACGCCTGG
WIN1	Forward	ACGTTAATGTCCAAATCTAGTCCGCCG
(PGSC003DMT400050017)	Reverse	TCCTTACCTGAACGCCTGTCA
Ef1-alpha	Forward	GATTGGAAACGGATATGCTCC
(PGSC0003DMT400059832)	Reverse	TCCTTACCTGAACGCCTGTCA

Potatoes harvested in fall of 2017 were treated with DMN at endo-dormancy, placed in storage at 8°C, and 90% RH, and retreated with DMN at early eco-dormancy. RNA was isolated from early eco-dormant tubers as described in the *Quick*-RNA Plant Miniprep Kit (www.zymoresearch.com), resulting in replicating expression analysis for PR-4/WIN2 to a third harvest year. Briefly, remaining biological duplicates (2 control, 2 DMN-treated) were retrieved from -80°C storage, subjected to bead beating, and column filtered to isolate RNA. First strand cDNA was produced using Superscript IV reverse transcriptase (www.thermofisher.com) with oligo-dT primers. Gene expression was determined with qRT-PCR using Fast SYBR Green Master Mix (Applied Biosystems, www.thermofisher.com) and the ddCt method [14] which provides the log fold difference in expression. The reference gene or endogenous control used was *EF1*-α. Three technical replicates were averaged using the StepOne Real-Time PCR System (Thermofisher.com) software to produce dCt means and standard errors, as well as ddCt values. After ddCt values were converted to fold changes in expression [14], biological replicates were averaged and 95% confidence intervals were calculated in Microsoft Excel for Mac 2011 (www.microsoft.com).

## Results

Treatments involved the use of 0.32 μl DMN per liter of chamber head space and total tuber weights of 643.02±29.4 g. The DMN residue levels on tubers was < 0.01 ppm for controls and 3.5±0.6 ppm, which reflects the levels used in the commercial sheds for tuber storage. There was no significant difference between the amount and quality of RNA isolated from control or DMN treated tuber tissue demonstrating that the application of the sprout inhibitor did not alter RNA stability or alter global stability of cellular transcripts.

### RNA-seq

**Fig 1:**
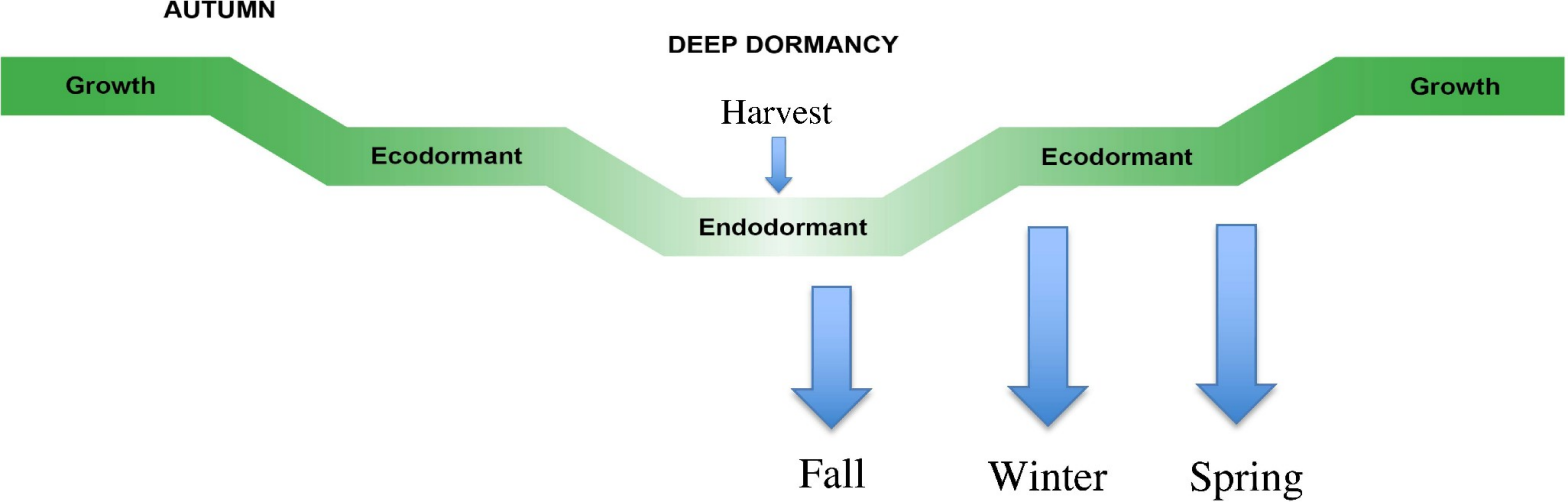
Treatment protocol for tubers harvested in two different years. Two weeks after harvest endo-dormant tubers were treated with DMN (Fall), RNA samples were collected for sequencing from a subset and the remaining tubers were placed under commercial storage conditions until tubers exited the endo-dormant state and entered early eco-dormancy (Winter). Tubers were retreated with DMN and transcript analysis was then conducted on a subset; the remaining tubers returned to storage. In early spring the tubers were retreated with DMN and RNA was then isolated for expression analysis (late eco-dormancy).

RNA samples were taken at three different developmental stages (Fig 1). Two weeks after harvest tubers were in deep dormancy (endo-dormant). After storage of about three months the meristems (tuber eyes) began to show an increase in size but growth was inhibited by the cold storage temperature (early eco-dormant). Further storage (five months) resulted in a complete loss of endodormancy (late eco-dormant). DMN treatments were applied at the endo-dormant state (Fall, two weeks after harvest), the first eco-dormant state (winter, three months after harvest), and after prolonged storage (spring, five months after harvest).

The total number of raw reads and mapped reads for each sample are presented in Table 2.

**Table 2:** Total raw reads and mapped quality reads for all samples.

Season 1 DMN Illumina Sequencing	Total Raw Reads	Mapped Reads	Season 2 DMN Illumina Sequencing	Total Raw Reads	Mapped Reads
Cont_Early_Ecodorm1_yr1	14666508	5787874	Cont_Endodorm1_yr2	27431034	9508808
Cont_Late_Ecodorm1_yr1	9772083	3632527	Cont_Early_Ecodorm1_yr2	28851459	8983346
Cont_Endodormt1_yr1	11278668	4569311	Cont_Early_Ecodorm2_yr2	25113207	9290948
Cont_Early_Ecodorm2_yr1	8103026	2393086	Cont_Late_Ecodorm1_yr2	28591017	8660093
Cont_Late_Ecodorm1_yr1	12619665	3986224	Cont_Late_Ecodorm2_yr2	33428159	10828091
Cont_Endodorm2_yr1	7797930	1999349	DMN_Endodorm1_yr2	27694771	7827080
Cont_Early_Ecodorm3_yr1	11747166	3945938	DMN_Early_Ecodorm1_yr2	25666052	7269738
Cont_Late_Ecodorm3_yr1	6543247	2385485	DMN_Early_Ecodorm2_yr2	26986998	8577345
Cont_Endodorm3_yr1	8243036	3136583	DMN_Late_Ecodorm1_yr2	28412227	8704171
Cont_Early_Ecodorm4_yr1	8352964	2747011	DMN_Late_Ecodorm2_yr2	32199014	7569540
Cont_Late_Ecodorm4_yr1	10499502	3092301	Total_Reads_yr2	284373938	87219160
Cont_Endodormant4_yr1	15125643	1386764			
DMN_Early_Ecodorm1_yr1	6371699	2495273			
DMN_Endodorm1_yr1	9009549	3378244			
DMN_Late_Ecodorm1_yr1	10528978	3420069			
DMN_Endodorm2_yr1	12623712	3617767			
DMN_Early_Ecodorm2_yr1	9252453	3087721			
DMN_Late_Ecodorm2_yr1	7856438	2303301			
DMN_Endodorm3_yr1	8646511	3347687			
DMN_Early_Ecodorm3_yr1	4898912	1866504			
DMN_Late_Ecodorm3_yr1	7303100	2510091			
DMN_Endodorm4_yr1	8805422	2417988			
DMN_Early_Ecodorm4_yr1	20933916	6647645			
DMN_Late_Ecodorm4_yr1	7804805	2199453			
Total_Reads_yr1	238784933	76354196			

The number of sample replicates varied, with four replicates for year one, and two replicates for each sample in year two. Given that the total number of reads was similar between years, the depth of sequencing per sample varied between the two years. Despite this level of comparison, the percent of the genome responding to DMN treatment in years one and two, regardless of dormancy status, was in the narrow range from 16.6 to 18.4 percent of the unique RNAs mapped.

### Expression differences

**Fig 2:**
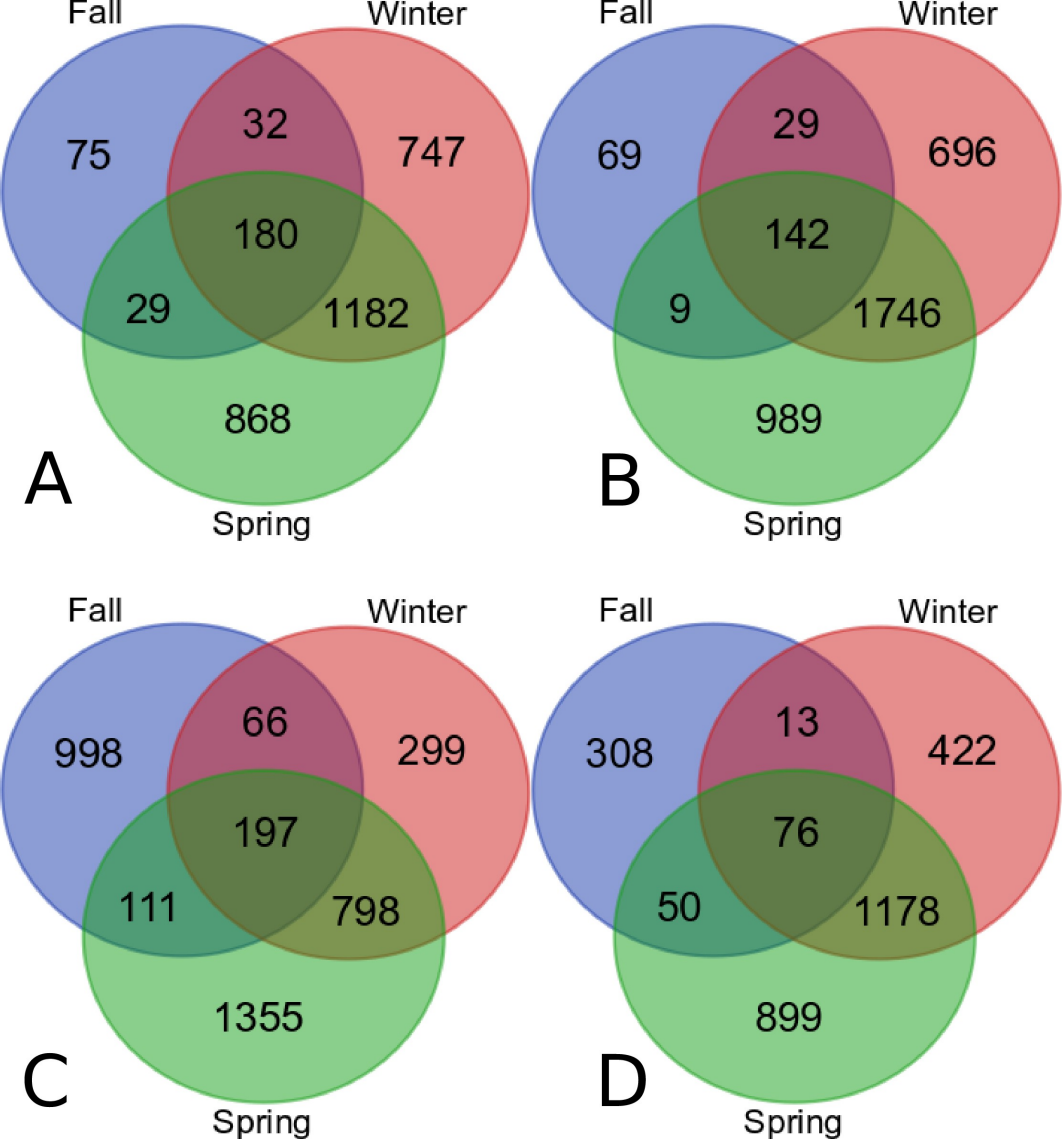
Venn diagram showing the overall statistically significant transcript changes in response to DMN. Changes in transcript levels in year one that are represented as up-regulated (A) or down-regulated (B). Changes in transcript levels in year two are represented as up-regulated (C) or down-regulated (D).

Gene expression changes were found to occur as tubers progressed during storage from the endo-dormant state to the eco-dormant state initiated in winter and spring and significant differences in expression for season one and season two are found in S1 and S2 Tables, respectively. Statistically significant changes induced by DMN (q< 0.05) resulted in 3113 transcripts up-regulated and 3680 down-regulated transcripts in 2015. Similar expression changes were found in 2016 with DMN inducing 3824 transcripts and suppressing 2946 transcripts. Changes induced by DMN, regardless of storage time or dormancy status, involved the upregulation of 180 transcripts in 2015 and 197 transcripts in 2016, while down regulated transcripts were 142 in 2015 and 76 in 2016 (Fig 2).

Gene set enrichment using the topGO package facilitated the parsing of analysis to gene set responses in endo-dormancy, early eco-dormant and late eco-dormant meristems. DMN repressed four biological processes in endo-dormant meristems, 27 processes in early eco-dormant, and 32 processes in late eco-dormant meristems (S3 Table). Biological processes induced by DMN were two for endo-dormant, 14 for early eco-dormant, and 15 for late eco-dormant. The difference in the number of biological processes changed by DMN suggests that sensitivity to DMN increases with time in storage and was dependent on tuber age. Down regulation of biological processes associated with the regulation of cell replication and division (GO:0000911, GO:0008283, GO:0007018, GO:0051301, GO:0000910, GO:0000226) first appear in early eco-dormancy and all, except G0:0000910, are also repressed by DMN in late eco-dormancy. DMN decreased the expression of multiple GO processes associated with epigenetic responses and DNA methylation (GO:0006303 DNA methylation, GO:0051567 histone H3-K9 methylation, GO:0006346 methylation-dependent chromatin silencing, GO:0010424 DNA methylation on cytosine within a CG sequence, G):0043987 histone H3-S10 phosphorylation, GO:0006342 chromatin silencing).

A smaller number of biological processes are induced by the exposure to DMN (S4 Table). Only two biological processes are up-regulated in endo-dormant meristems: GO:0015967 (diadenosine tetraphosphate catabolic process) and GO:0006857 (oligopeptide transport). Once endo-dormancy is terminated 14 biological processes are induced in early eco-dormancy while 15 are induced in later eco-dormancy. The process of systemic acquired resistance via salicylic acid (GO:0009862) is induced in both early and late eco-dormancy. Induction of transcripts associated with salicylate signaling response and processing are induced in early eco-dormancy tubers (GO:0009751) and late eco-dormancy (GO:0009863). Transcripts associated with biological processes involving jasmonic acid synthesis, signaling, and response (GO:0009867, GO;0009753, GO:0009695) are induced by DMN in either early or late eco-dormancy. In the eco-dormant state there is also upregulation of biological processes associated with stress (GO:0006950) and defense responses (GO:0010363, GO:0052544, GO:0009816, GO:0050832, GO:0031348, GO:0010200) in addition to processes involved with heat (GO:0009408) and drought (GO:OOO6972).

A gene set encoding for wound-inducible/PR-4 proteins found on chromosome 1 was up-regulated following DMN exposure. This gene set exhibited a greater response to DMN as tubers aged. However, mapping of RNA to this region became somewhat problematic in that expression levels were derived for each region (PGSC003DMT400050017 and PGSC003DMT500050018) but some RNAs mapped across the entire region because of assignment to a larger DNA fragment found in the annotation file. To clarify the expression for this gene set, qRT-PCR was conducted on tubers harvested in a third year (Table 3).

**Table 3:** qRT-PCR results using biological duplicates with technical triplicates.

Gene	Control, DMN-Treated	ΔCT Mean	ΔCT	ΔΔCT	2-ΔΔCT	Expression Fold Change (95% CI)
Win1_Rep 1	36.8, 32.06	9.98, 4.17	0.19, 0.17	0.00, -5.81	1.00, 56.17	
Win1_Rep 2	36.49, 32.06	9.63, 3.97	0.26, 0.15	0.00, -5.65	1.00, 50.3	1.00, 53±5.87
Win2_Rep 1	25.49, 21.69	-8.96, -14.77	0.16, 0.25	0.00, -5.82	1.00, 56.37	
Win2_Rep 2	29.53, 24.91	-5.01, -11.03	0.07, 0.08	0.00, -6.01	1.00, 64.52	1.00, 60.45±8.15

Using the ddCt method it was determined that in early eco-dormant tubers, both *WIN1* and *WIN2* (from the PR-4/WIN2 region on chromosome 1) transcripts were increased with DMN treatment (5.73201 and 5.914285 respectively). These results confirm the expression difference data from the RNA-seq experiment.

DMN repressed four biological processes in endo-dormant meristems, 27 processes in early eco-dormant, and 32 processes in late eco-dormant meristems (S2 Table). Biological processes induced by DMN were two for endo-dormant, 14 for early eco-dormant, and 15 for late eco-dormant. The difference in the number of biological processes changed by DMN suggests that sensitivity to DMN increases with time in storage and was dependent on tuber age. Down regulation of biological processes associated with the regulation of cell replication and division (GO:0000911, GO:0008283, GO:0007018, GO:0051301, GO:0000910, GO:0000226) first appear in early eco-dormancy and all, except G0:0000910, are also repressed by DMN in late eco-dormancy.

A homolog for AtDi19 (drought-induced) was found in the potato genome (PGSC0003DMT400063764). The log2 fold change for this gene was not significantly different in endo-dormant tubers treated with DMN but it was induced by DMN in early and late eco-dormancy. AtDi19 has been shown to a drought-induced transcription factor that induces the expression of pathogenesis-related (PR) proteins [15]

Analysis of RNA-seq data showed a gene cluster encoding for osmotin/PR-5 transcripts located on chromosome 8 was induced by exposure to DMN. The cluster was composed of five tandemly arranged regions that showed DMN induction was much greater as tubers aged in storage and transitioned from the endo-dormant state to an eco-dormant state. No RNA sequences mapped to the second gene in the cluster suggesting that it may be nonfunctional. This gene was the only one within the cluster to have intronic regions.

## Discussion

Previous studies on dormant tubers demonstrated that DMN increased the expression of transcripts that encode for KRP-like proteins [16], which are known suppressors of cell proliferation [17]. RNA-seq data in the current study did reveal that DMN induced PGSC0003DMT400019919, a CDK-inhibitor and a KRP-like protein, but the changes in expression were only significant (q< 0.05) in winter and spring treatments. This suggests that there is a correlation between DMN suppression of proliferation and the eco-dormant state of harvested tubers. The natural suppressed state of cell division, associated with endodormancy, does not appear to respond to DMN upregulation of a gene associated with CDK inhibition.

Across both years, and during all stages of dormancy and storage, 26 transcripts (q < 0.05) were downregulated by DMN (S3 Table), while 57 transcripts were induced by DMN (S2 Table). Assigning a gene ontology biological process to the down-regulated transcripts showed activities such as auxin signaling, meristem function, as well as cellulose and lignin biosynthesis were affected, suggesting a link to the growth suppression induced by DMN. The biological process of transcripts induced by DMN included membrane and lipid transport, and responses to multiple stressors including oxidative conditions and pathogens, suggesting that DMN is inducing a global response to stress. However, there appears to be no linkage of the induced processes in any stage of eco-dormancy to the direct control and regulation of cell growth and division. The suppression of a number of GO defined biological processes linked to histone modification or DMNA methylation suggests that gene expression changes induced by DMN maybe a result of an epigenetic response.

**Fig 3:**
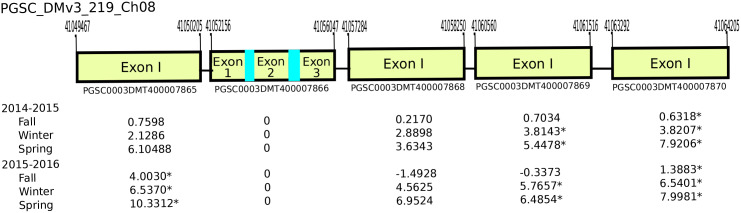
Expression of osmotin/PR5-like gene cluster found on chromosome 8. All genes show an increase in expression as tubers age except the second gene in the series that encodes for the transcript PGSC0003DMT400007866. All values are given as FPKM and those marked with * indicate a q-value of < 0.05.

DMN had a notable induction of gene clusters encoding for PR-4-like and PR-5-like proteins. In plants the pathogenesis-related proteins are induced by biotic and abiotic stress [15, 18, 19]. PR5, or osmotin-like proteins, were first described because of their induction in tobacco plants exposed to increased osmotic stress [20]. The up-regulation by DMN of the potato homolog for the Arabidopsis transcription factor Di19 suggests a mechanism for the induction of the PR5 gene cluster chromosome 8 (Fig 3). PR5 has been shown to be induced in response to salt and drought stress [21]. The results from Gene Ontology analysis demonstrate gene networks linked to SA are increased in response to DMN and previous research has shown that SA and Jasmonic acid alter the expression of PR proteins [22]. Thus, DMN may be functioning through this SA signaling mechanism in potato tubers to induce the drought associated protein PR5.

Wound-induced genes *WIN1* and *WIN2*, part of a multi-gene family in potatoes, were first described by Stanford [23], and as such were shown to exhibit differential expression in various tissues of a potato plant following wounding. *WIN1* and *WIN2* are organized in tandem and encode nearly identical polypeptides. Their coding sequences were found to be highly homologous to each other (81%), with *WIN2* encoding a polypeptide of 211 amino acids and *WIN1* with 200, including a C-terminal 25 amino acid signal peptide that was later found to be conserved among Class I PR-4 genes [24]. High homology to other plant defense proteins was noted, including to hevein, chitinase, and several lectins that all align over the hevein domain [23, 25]. This sugar-binding hevein domain at the N-terminal of *WIN1* and *WIN2* was hypothesized to recognize or bind chitin. In response to wounding, Stanford [23] discovered that RNA specific *WIN1* accumulated in wounded leaves and stems but was not detected in roots and tubers, while *WIN2*-specific RNA was found in leaves, stems, tubers, and roots. *WIN2* was studied further [26] in transgenic potato plants and it was found to respond to mechanical wounding, first in cells next to the wound and later in cells of the vascular system. In tubers specifically, only a localized response to a wound was present unless other areas of the tuber had begun to sprout, indicating an active vascular transport system was needed to extend the wound response of *WIN2* and that the tuber developmental stage may influence *WIN2* regulation [26].

As cross-hybridization among PR-4 Class I probes, as well as PCR primers designed for one particular protein but based on the sequence of another, has been found consistently [23, 27, 28], this study included the design of primer pairs specific to *WIN1* and to *WIN2*, that cross each transcript’s intron region to definitively determine, via the comparative ddCt qRT-PCR, which gene is being upregulated in response to DMN treatment.

**Fig 4:**
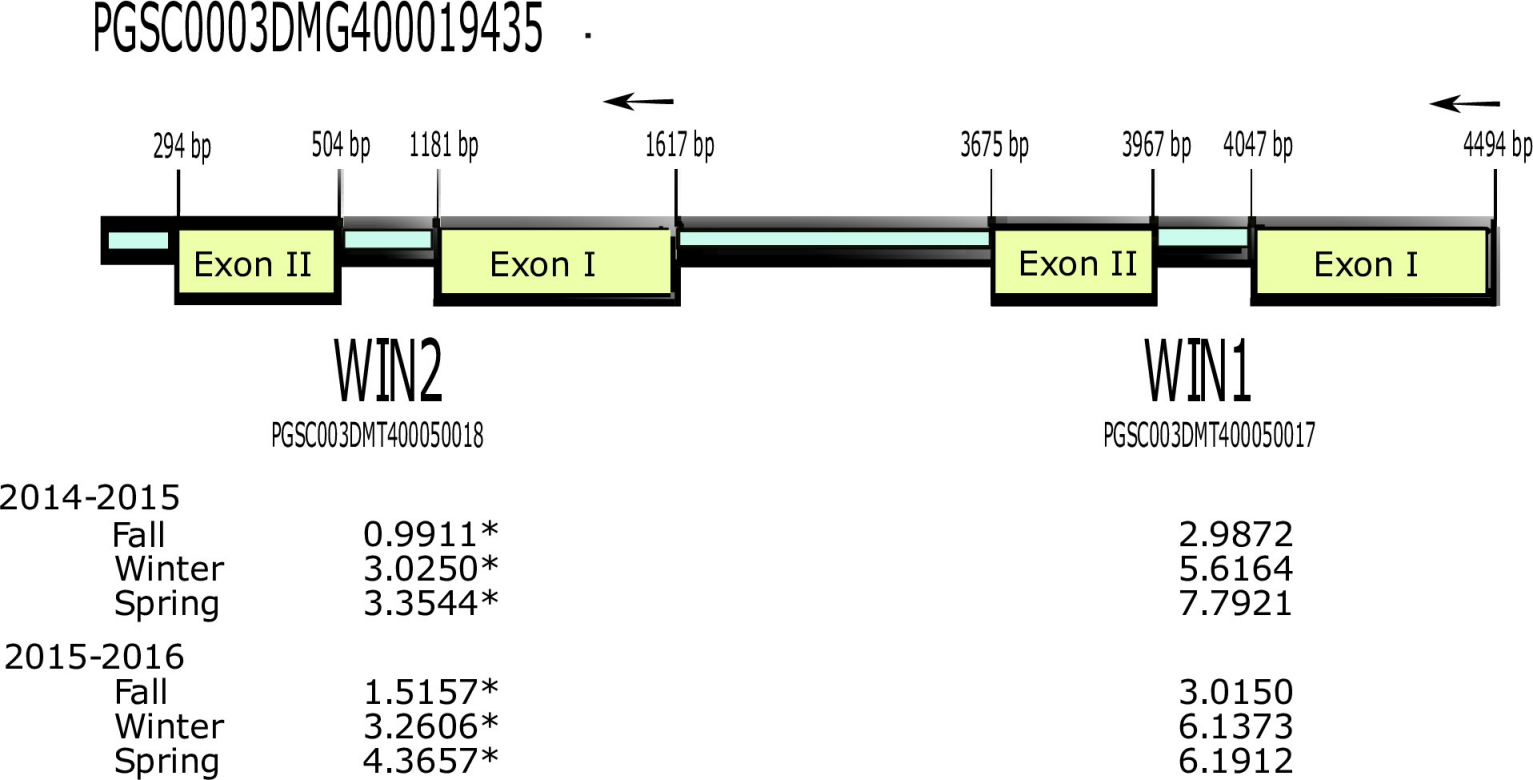
Expression of the WIN/PR-4-like gene set found on chromosome 1. All values are given as FPKM and those marked with * indicate a q-value of < 0.05.

In DMN-treated tubers, both *WIN1* and *WIN2* were upregulated, however only the latter was statistically significant (Fig 4). This was expected as in the first study of WIN proteins, *WIN1* was detected in potato stems and leaves, but not roots and tubers after wounding, but *WIN2* was found to accumulate in all tissues examined [23]. Additional experiments with transgenic potatoes revealed that sprouting tubers with a vascular system produced an extended response, ie. *WIN2* upregulation, beyond the wound-adjacent cells [26]. Thus, we would expect that meristems themselves would show a significant upregulation of *WIN2* with proper stimulation, in this case, DMN treatment. DMN was first discovered in stored tubers only in the dormant state, suggesting that it plays a role in the regulation of plant growth and it may be through this regulation that *WIN2* expression is modified.

PR-4 Class I proteins have been shown to act independently of salicylic acid [29] but are often induced by pathogens via jasmonic acid (JA) dependent pathways [29] as methyl jasmonate [27, 28, 30]. It is thus possible that DMN may mimic a member of the JA pathway, or influence *COI1*, the receptor gene for active JA [29, 31]. Perhaps, likely acting as a signal molecule suggesting that DMN can be added to the list of known systemic acquired resistance chemicals that induce basic PR proteins and wound-associated genes [32]. The mode of action of DMN may also involve an epigenetic response functioning through changes in DNA methylation and/or histone modification systemic acquired resistance chemicals that induce basic PR proteins and wound-associated genes.

## Supporting information

S1 Table (XLSX)

S2 Table (XLSX)

S3 Table (XLSX)

S4 Table (XLSX)
